# The Novel Deacetylase Inhibitor AR-42 Demonstrates Pre-Clinical Activity in B-Cell Malignancies *In Vitro* and *In Vivo*

**DOI:** 10.1371/journal.pone.0010941

**Published:** 2010-06-03

**Authors:** David M. Lucas, Lapo Alinari, Derek A. West, Melanie E. Davis, Ryan B. Edwards, Amy J. Johnson, Kristie A. Blum, Craig C. Hofmeister, Michael A. Freitas, Mark R. Parthun, Dasheng Wang, Amy Lehman, Xiaoli Zhang, David Jarjoura, Samuel K. Kulp, Carlo M. Croce, Michael R. Grever, Ching-Shih Chen, Robert A. Baiocchi, John C. Byrd

**Affiliations:** 1 Department of Internal Medicine, The Ohio State University, Columbus, Ohio, United States of America; 2 Department of Molecular Virology, Immunology, and Medical Genetics, The Ohio State University, Columbus, Ohio, United States of America; 3 Department of Molecular and Cellular Biochemistry, The Ohio State University, Columbus, Ohio, United States of America; 4 College of Pharmacy, The Ohio State University, Columbus, Ohio, United States of America; 5 Center for Biostatisics, Comprehensive Cancer Center, The Ohio State University, Columbus, Ohio, United States of America; University of Barcelona, Spain

## Abstract

**Background:**

While deacetylase (DAC) inhibitors show promise for the treatment of B-cell malignancies, those introduced to date are weak inhibitors of class I and II DACs or potent inhibitors of class I DAC only, and have shown suboptimal activity or unacceptable toxicities. We therefore investigated the novel DAC inhibitor AR-42 to determine its efficacy in B-cell malignancies.

**Principal Findings:**

In mantle cell lymphoma (JeKo-1), Burkitt's lymphoma (Raji), and acute lymphoblastic leukemia (697) cell lines, the 48-hr IC_50_ (50% growth inhibitory concentration) of AR-42 is 0.61 µM or less. In chronic lymphocytic leukemia (CLL) patient cells, the 48-hr LC_50_ (concentration lethal to 50%) of AR-42 is 0.76 µM. AR-42 produces dose- and time-dependent acetylation both of histones and tubulin, and induces caspase-dependent apoptosis that is not reduced in the presence of stromal cells. AR-42 also sensitizes CLL cells to TNF-Related Apoptosis Inducing Ligand (TRAIL), potentially through reduction of c-FLIP. AR-42 significantly reduced leukocyte counts and/or prolonged survival in three separate mouse models of B-cell malignancy without evidence of toxicity.

**Conclusions/Significance:**

Together, these data demonstrate that AR-42 has *in vitro* and *in vivo* efficacy at tolerable doses. These results strongly support upcoming phase I testing of AR-42 in B-cell malignancies.

## Introduction

Deacetylases (DACs) are a family of enzymes that catalyze the removal of acetyl groups from lysine residues, and to date have been extensively studied in the context of histone proteins. Inhibitors of these enzymes were originally reported to relieve transcriptional repression and subsequent epigenetic silencing caused by histone deacetylation. It is now evident that the targets of these enzymes also include a broad array of proteins such as transcription factors, chaperones, signaling components, and cytoskeletal proteins. Thus, the effects of DAC inhibitors are diverse and incompletely understood, and likely vary by cell type and context. Adding to the complexity of reported DAC inhibitor activities is the different, but occasionally overlapping, effects on class I and II DACs. Class I DACs (1, 2, 3 and 8) are primarily found in the nucleus, although DAC3 is found in both the nucleus and cytoplasm. Class II DACs (4, 5, 6, 7, 9 and 10) are generally reported to shuttle in and out of the nucleus, depending on intracellular signals. DAC6 is a cytoplasmic enzyme that deacetylates tubulin [1], HSP90 [2], [3], and likely additional cytoplasmic proteins. Due to their broad effects on gene transcription, cell growth and differentiation, inhibitors of DACs have been shown to possess anti-cancer activity in a variety of tumor cell models, in primary tumor cells, and *in vivo* [4], [5], [6]. Clinical efficacy of this class of agents to date is perhaps best exemplified by vorinostat (SAHA) and romidepsin (depsipeptide; FK228) in cutaneous T-cell lymphoma, in which response rates of approximately 30–35% are noted. However an enormous body of evidence also supports the investigation of this class of agents in tumors as diverse as prostate cancer, lung cancer and glioblastoma [4], [7], [8].

Chronic Lymphocytic Leukemia (CLL) is immunophenotypically defined as a malignancy of CD5/CD19/CD23 positive, CD20 and Ig dim B cells that manifests with bone marrow failure, lymphadenopathy and infections as a consequence of disease-associated immune suppression. While recent advances in chemoimmunotherapy strategies have improved options for CLL patients, the median overall survival for fludarabine-refractory patients is just 13 months. Mantle cell lymphoma (MCL), an aggressive B cell malignancy, is characterized by the abnormal proliferation and accumulation of CD5/CD20/CD22 positive, CD23 negative B cells in various hematopoietic tissues, with or without peripheral blood involvement. While MCL comprises approximately 8% of Non-Hodgkin lymphoma cases, it is associated with a disproportionate number of deaths and a mean survival of only three years [9]. To date, therapeutic options for these two B cell diseases are limited, and relapses are nearly universal. Given the absence of effective therapies for these and other B-cell malignancies, it is essential to explore new treatment options.

Multiple studies have demonstrated that DAC inhibitors including romidepsin, entinostat (MS-275) and valproic acid can alter histone acetylation status in CLL and lead to selective cytotoxicity in these cells [10], [11], [12], [13]. In preclinical studies done by our group, the class I DAC inhibitor romidepsin induced apoptosis in CLL cells via activation of caspase 3 and caspase 8, with minimal alteration in caspase 9 activity [10]. Caspase 8 activation occurred concomitantly with down-regulation of c-FLIP, an inhibitory protein of caspase 8. The observation that romidepsin operates via a caspase 8-mediated process is significant, as this pathway is not typically activated by other agents currently used in the treatment of CLL. Subsequent work by our group has demonstrated that entinostat, also a class I-specific DAC inhibitor, promotes apoptosis in CLL cells with concurrent alteration in lysine acetylation of histones H3 and H4 [14]. Whereas others report that entinostat and other DAC inhibitors may mediate their cytotoxicity through generation of reactive oxygen species, we demonstrated that this occurred later in the process of CLL cell death, and was likely an effect rather than a cause [14].

Clinical trials with class I DAC inhibitors *N*-acetyldinaline (CI-994; J Byrd, personal communication), romidepsin [11] and MGCD0103 [15] have been performed in CLL, with the former two demonstrating evidence of anti-tumor activity as supported by improvement in lymphocyte counts and diminishment in lymph node size. No significant clinical activity was observed with MGCD0103 in CLL [15]. In all three of these trials, significant fatigue, anorexia, and other constitutional symptoms limited compliance and patient willingness to continue therapy. To date, clinical testing of class I/II DAC inhibitors in CLL has been extremely limited.

AR-42 (previously called OSU-HDAC42) is a novel hydroxamate-tethered phenylbutyrate derivative [16], [17] with *in vitro* and *in vivo* activity in multiple solid tumor models [18], [19], [20], [21] and more recently, in mouse and canine mast cells [22]. AR-42 was demonstrated to be more potent than the benchmark agent vorinostat in inducing apoptosis and in causing reductions of phospho-Akt, Bcl-xL, and survivin. *In vivo*, AR-42 suppressed PC-3 tumor xenograft growth by 67%, whereas vorinostat at the same dose suppressed growth by 31% [19]. Based on our previous studies of class I DAC inhibitors in CLL and these encouraging results, we tested AR-42 *in vitro* and *in vivo* using CLL and related B-cell malignancies.

## Results

### *In vitro* activity of AR-42

**Figure 1:**
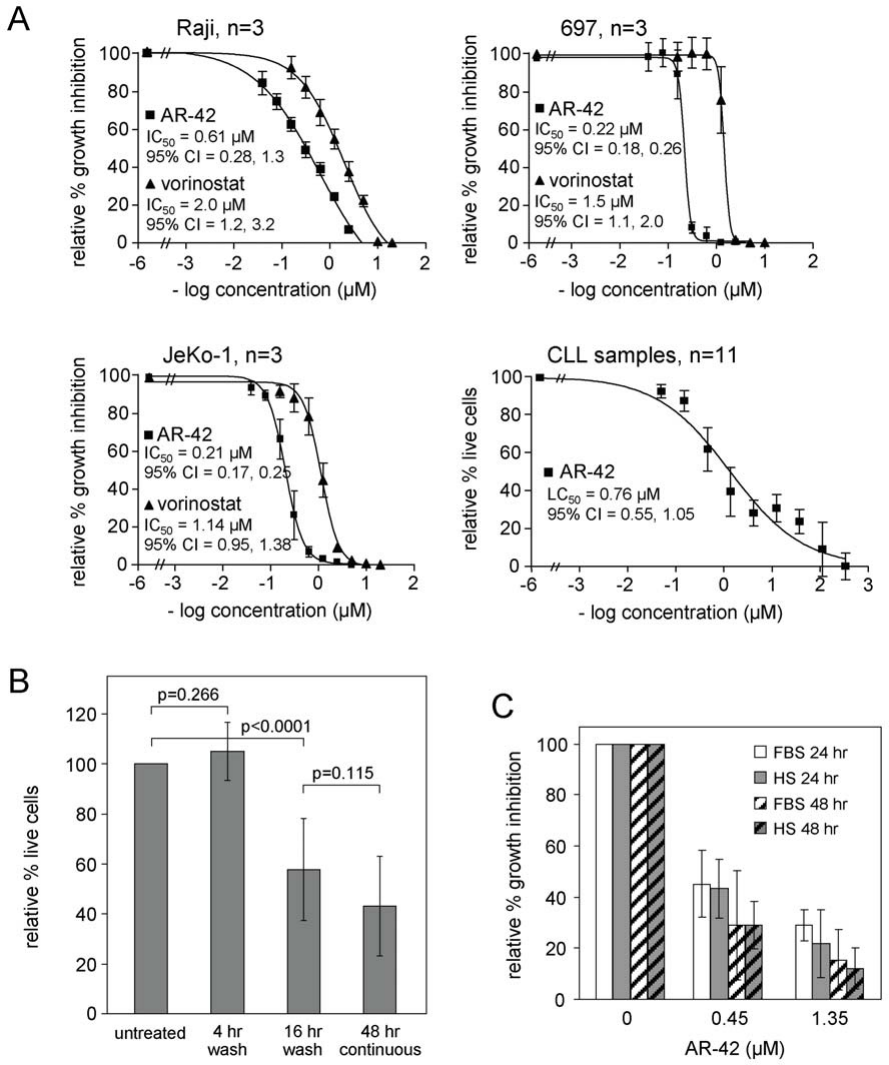
AR-42 exhibits potent cytotoxicity in B-leukemia/lymphoma cells. (**A**) Raji, 697 and JeKo-1 B-cell lines (n = 3 each) or CLL patient cells (n = 11) were incubated 48 hr with or without AR-42. Growth inhibition (for cell lines) or viability (for non-proliferating CLL patient cells) was assessed by MTT assay and calculated relative to time-matched untreated cells. Bars represent +/− standard deviation. (**B**) CLL cells were incubated with or without AR-42 (0.90 µM). At 4 hr or 16 hr, cells were replated in fresh media with or without drug. At 48 hr, viability was assessed by MTT assay and is shown relative to time-matched, untreated controls (n = 5 for 4 and 16 hr exposures; n = 11 for 48 hr exposure and untreated controls). Bars represent +/− standard deviation. There was no significant difference in viability between untreated samples and samples with 4 hr AR-42 exposure. The difference in viability between untreated samples or those with 16 hr exposures was significant (p<0.0001). (**C**) 697 cells were incubated 24 hr (solid bars) or 48 hr (striped bars) with or without AR-42, using RPMI 1640 supplemented either with 10% FBS (white bars) or 10% human serum (grey bars) (n = 3). Viability was assessed by MTT assay and calculated relative to the untreated samples. Bars represent +/− standard deviation. AR-42 cytotoxicity in media with human serum or FBS was similar (0.45 µM: p = 0.20 and p = 1.0 at 24 and 48 hr respectively; 1.35 µM: p = 0.21 and p = 0.24 at 24 and 48 hr, respectively).

In MTT assays (**figure 1A**), the 50% growth inhibitory concentration (IC_50_) of AR-42 at 48 hr was 0.61 µM (95% CI = 0.28, 1.33) in Raji Burkitt's lymphoma cells, 0.22 µM (95% CI = 0.18, 0.26) in 697 acute lymphoblastic leukemia cells, and 0.21 µM (95% CI = 0.17, 0.25) in JeKo-1 MCL cells (n = 3 each). In simultaneous assays, the IC_50_ values of vorinostat were 3- to 6-fold higher, consistent with results in prostate cancer cell lines [19]. In CLL patient cells, AR-42 exhibited a 48-hr LC_50_ of 0.76 µM (95% CI = 0.55–1.05; n = 11), similar to what we observed with the class I DAC inhibitor entinostat (MS-275) [14]. For the *in vitro* work presented here, AR-42 was used at the LC_50_ of 0.90 µM that was initially calculated using a smaller number of CLL samples. Although this LC_50_ was found to be moderately lower when additional CLL samples were included, we continued to use the initial LC_50_ of 0.90 µM for consistency among experiments.

In washout experiments using CLL tumor cells, the 48-hr cytotoxic effect of AR-42 was eliminated when the drug was removed after 4 hr (n = 5; p = 0.266 relative to the untreated control). However, cytotoxicity with a 16 hr exposure was similar to that observed when samples were incubated continuously for 48 hr (n = 5; p = 0.115 relative to continuously treated cells) (**figure 1B**). We previously observed that the cytotoxic activity of the cyclin-dependent kinase inhibitor flavopiridol was substantially reduced in medium containing human serum vs. fetal bovine serum, with profound implications for effective clinical administration [23]. We therefore compared the cytotoxicity of AR-42 against 697 cells incubated in RPMI 1640 media supplemented with 10% human serum or 10% fetal bovine serum. AR-42 showed no difference in cytotoxicity between these two serum conditions (p≥0.20 at 24 and 48 hr; **figure 1C**).

**Figure 2:**
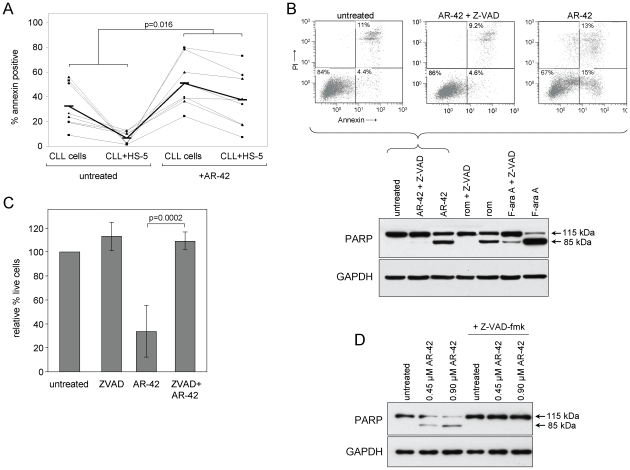
AR-42 efficacy is independent of stromal survival factors but involves caspase activation. (**A**) CLL patient cells (n = 8) were incubated 16 hr with or without AR-42 (0.90 µM), then washed and cultured in the absence or presence of HS-5 stromal cells for a total of 48 hr. CLL cells were collected and analyzed by flow cytometry. Data for each patient are shown individually (light dotted lines) as absolute percent annexin-positive cells. Averages for each group are shown as a dark bar, connected by a solid line. The difference in HS-5 protection of CLL cells between untreated and AR-42 treated was significant (p = 0.016). (**B**) JeKo-1 cells (n = 3) were incubated 24 hr with or without AR-42 (0.90 µM), romidepsin (rom; 0.04 µM), F-ara A (5.0 µM) and Z-VAD-fmk (100 µM). Cells were analyzed by annexin/PI flow cytometry (top) or by immunoblot for PARP (bottom). Results with 697 cells were similar. (**C**) CLL cells (n = 5) were incubated with or without AR-42 (0.90 µM) and Z-VAD-fmk (100 µM). Cells were analyzed by flow cytometry at 48 hr and percent live cells (annexin and PI negative) were calculated relative to time-matched untreated samples. Bars represent +/− standard deviation. In AR-42 treated cells, increase in live cells with Z-VAD-fmk was significant (p = 0.0002). (**D**) Extracts from CLL cells incubated 24 hr with or without AR-42 and Z-VAD-fmk were immunoblotted for PARP. Result is representative of seven CLL samples.

CLL tumor cells are known to receive a variety of survival signals from the microenvironment, and cumulative evidence clearly demonstrates the importance of such signaling in CLL cell resistance to apoptosis and to chemotherapy [24], [25], [26]. We therefore investigated the efficacy of AR-42 in the presence of stromal protection using the human marrow-derived fibroblast cell line HS-5 [27]. HS-5 cells were seeded in tissue culture flasks one day prior to treatment. CLL patient cells (n = 8) were incubated with or without AR-42 (0.90 µM) 16 hr before washing and plating in flasks with or without HS-5 for a total of 48 hr. CLL cells were then recovered by gentle pipetting and analyzed by flow cytometry. Events due to non-adherent HS-5 cells were eliminated by forward/side scatter characteristics as determined by evaluation of HS-5 cells alone. Untreated CLL cells co-cultured with HS-5 cells showed dramatic reduction in apoptosis as measured by annexin positivity relative to non-co-cultured cells, as noted by a strong reduction the annexin-positive fraction (**figure 2A**). As expected, cells treated with AR-42 without HS-5 co-culture showed a substantial increase in annexin positivity at this time point. However, the degree of HS-5 protection was significantly different between untreated cells and cells treated with AR-42 (p = 0.016), indicating that the pro-survival effect of HS-5 is unable to effectively block AR-42-induced apoptosis. These results provide important evidence that AR-42 may circumvent the protective effects of the CLL cell microenvironment *in vivo*.

We performed additional experiments to clarify events accompanying AR-42 mediated cell death. Caspase activation and induction of the mitochondrial pathway of apoptosis are documented effects of most DAC inhibitors [14], [28]. However, Mitsiades *et al*. reported that caspases were not activated following vorinostat treatment in a myeloma cell line, nor did the broad caspase inhibitor Z-VAD-fmk protect these cells from vorinostat [29]. We therefore investigated the role of caspase activation in AR-42 mediated cell death in B-cell lymphoma lines. Cells were incubated 24 hr with 0.90 uM AR-42, with or without the broad caspase inhibitor Z-VAD-fmk (100 µM). AR-42-mediated apoptosis, as defined by annexin binding and processing of the caspase substrate polyADP ribose polymerase (PARP) to its 85 kDa form, was effectively abrogated by Z-VAD-fmk. Representative data from JeKo-1 are shown in **figure 2B**; similar results were obtained with 697 cells. We confirmed these results using CLL tumor cells treated with AR-42 in the presence or absence of Z-VAD-fmk. Relative to untreated controls, AR-42 caused a greater than 60% decrease in live (annexin and PI negative) cells at 48 hr, an effect that was nearly completely inhibited by Z-VAD-fmk (**figure 2C**, n = 5). AR-42 induced PARP cleavage in these same samples at 24 hr, which also was effectively prevented by Z-VAD-fmk (**figure 2D**, representative of 5 CLL patient samples).

### Class-specific activity of AR-42

**Figure 3:**
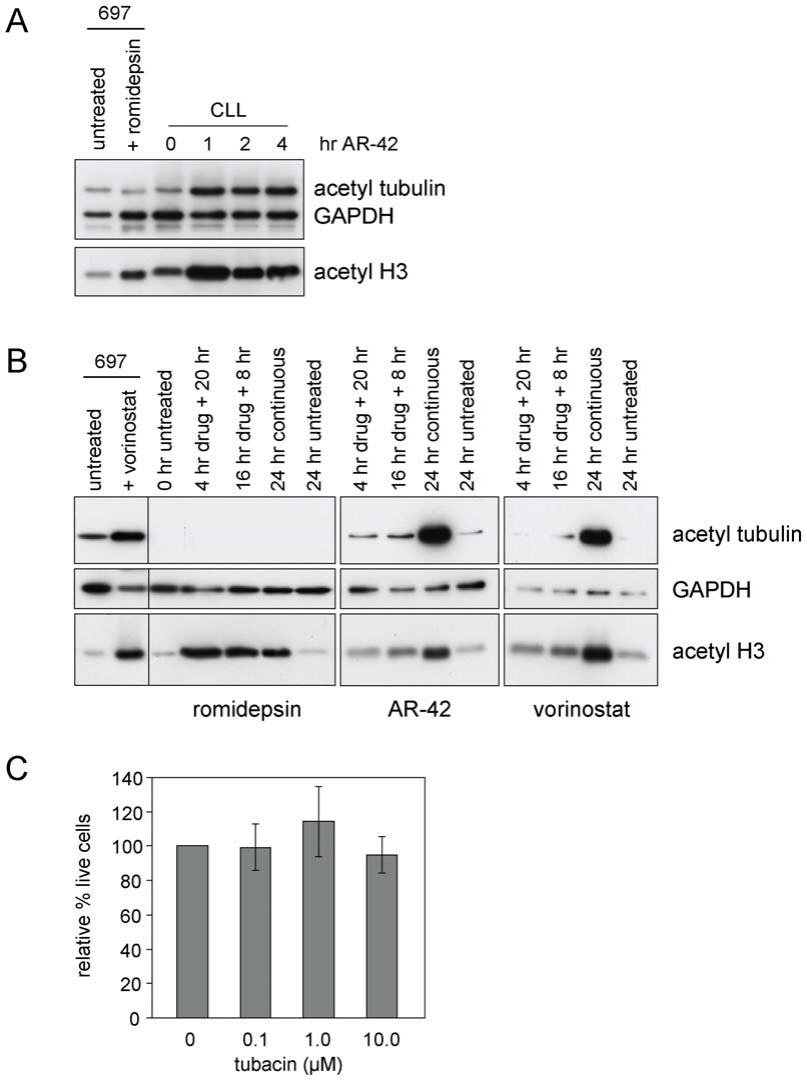
AR-42 exhibits class I and II DAC inhibitory activity. (**A**) CLL cells were incubated with or without AR-42 (0.90 µM) for the times noted. Lysates from 697 cells incubated 16 hr without or with 0.04 µM romidepsin were included as controls. Extracts were analyzed by immunoblot for acetylated tubulin and histone H3, plus GAPDH as a loading control. Data are representative of six patient samples. (**B**) CLL tumor cells were incubated with or without DAC inhibitors romidepsin (0.04 µM), AR-42 (0.90 µM), or vorinostat (5.0 µM) as indicated. 697 cells incubated 24 hr with or without 5.0 µM vorinostat were included as a control (left). Extracts were analyzed by immunoblot for acetylated tubulin and histone H3, plus GAPDH as loading control. Data are representative of seven CLL samples. (**C**) CLL patient cells (n = 4) were incubated without or with tubacin as indicated for 72 hr, and viability was analyzed by MTT assay. Data are shown relative to the time-matched untreated control. There were no significant changes in viability across doses (p = 0.265).

DAC inhibitory activity of AR-42 was assessed by examining acetylation of multiple downstream targets in CLL patient cells. In CLL patient cells, increased acetylation of class I DAC target histone H3 and the class II target tubulin could be detected with just 1 hr of exposure to 0.90 µM AR-42 (**figure 3A**, representative of six patient samples). After a 24 hour exposure, still prior to substantial cell death as determined by annexin/PI flow cytometry, AR-42-mediated increases in acetylation of H3 and tubulin in CLL cells were evident (**figure 3B**, representative of seven patient samples). In contrast, the class I-specific DAC inhibitor romidepsin produced no tubulin acetylation at this time point, although it is important to note that romidepsin and vorinostat concentrations were selected from previous work and do not represent equitoxic doses. Thus immunoblot results with these agents are presented for qualitative comparison only.

The DAC6-specific inhibitor tubacin [1] has been reported to have multiple effects on lymphoid cells attributable to DAC6 inhibition in addition to inducing acetylation of tubulin and HSP90. These include aggresome formation [30], motility [31], and cytotoxicity in EBV-positive lymphoma cells [32]. We therefore tested the effects of tubacin on CLL patient cells. No significant effects on cell viability, as measured by MTT assay, were noted at times up to 72 hr and concentrations up to 10 µM (**figure 3C**), suggesting that the tubulin and/or HSP90 deacetylation activity of DAC6 is not by itself crucial for CLL cell survival. However, these studies do not rule out a role for DAC6 inhibition in combination with inhibition of other DACs in promoting CLL cell death.

### AR-42 sensitizes CLL patient cells to apo2L/TRAIL

**Figure 4:**
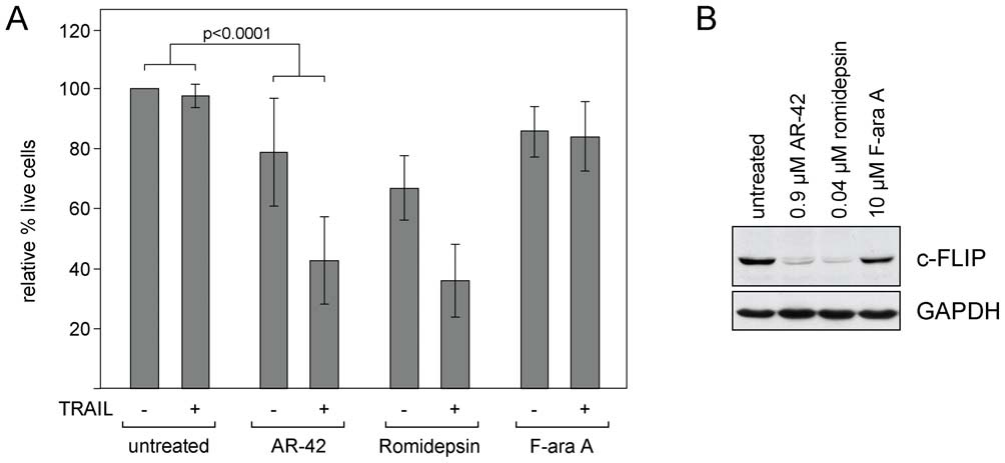
AR-42 sensitizes CLL cells to TRAIL and mediates reduction of c-FLIP. (**A**) CLL patient samples (n = 6) were incubated with or without AR-42 (0.90 µM) and TRAIL (100 ng/mL). Romidepsin (0.04 µM) and F-ara A (2.0 µM) were included as positive and negative controls, respectively. Cells were assessed by flow cytometry at 48 hr, and percent live (annexin and PI negative) cells were calculated relative to the time-matched untreated samples. Co-incubation with AR-42 and TRAIL produced a significant reduction in cell viability (p<0.0001) compared to TRAIL or AR-42 alone. (**B**) CLL cells were incubated 24 hr with or without AR-42, and lysates were analyzed for expression of c-FLIP. Romidepsin and F-ara A were included as positive and negative controls, respectively. Example is representative of seven CLL samples.

DAC inhibitors possessing class I inhibitory activity have shown the potential to sensitize many types of tumor cells [33], [34], including CLL [35], to tumor necrosis factor-related apoptosis inducing ligand (TRAIL). We therefore incubated CLL patient cells with or without AR-42 and recombinant TRAIL and examined the cells for apoptosis by annexin/PI flow cytometry. F-ara A (active form of fludarabine) was used as a negative control. AR-42 significantly increased the sensitivity of CLL cells to TRAIL (**figure 4A**, n = 6), as has been shown for class I DAC inhibitors such as romidepsin [35], [36]. We previously reported that romidepsin resulted in reduction of the caspase-8 inhibitory protein c-FLIP [10], potentially explaining sensitization to TRAIL as described by MacFarlane *et al*. [37]. We therefore investigated the effect of AR-42 on c-FLIP in CLL patient cells. As seen with romidepsin, AR-42 treatment of CLL cells resulted in notably reduced levels of c-FLIP by 24 hr (**figure 4B**, representative of seven CLL patient samples). This result was observed using a c-FLIP monoclonal antibody from Enzo Life Sciences as used in our earlier work [10], although no change in c-FLIP levels were noted using a polyclonal c-FLIP antibody (#556567, BD Pharmingen, San Diego CA) (data not shown). A similar discrepancy was also reported by Inoue *et al*. [36]. Therefore, in addition to cell type and inhibitor differences, reagent differences must also be considered when comparing these results with those of other publications [34], [36], [37].

### *In vivo* activity of AR-42

**Figure 5:**
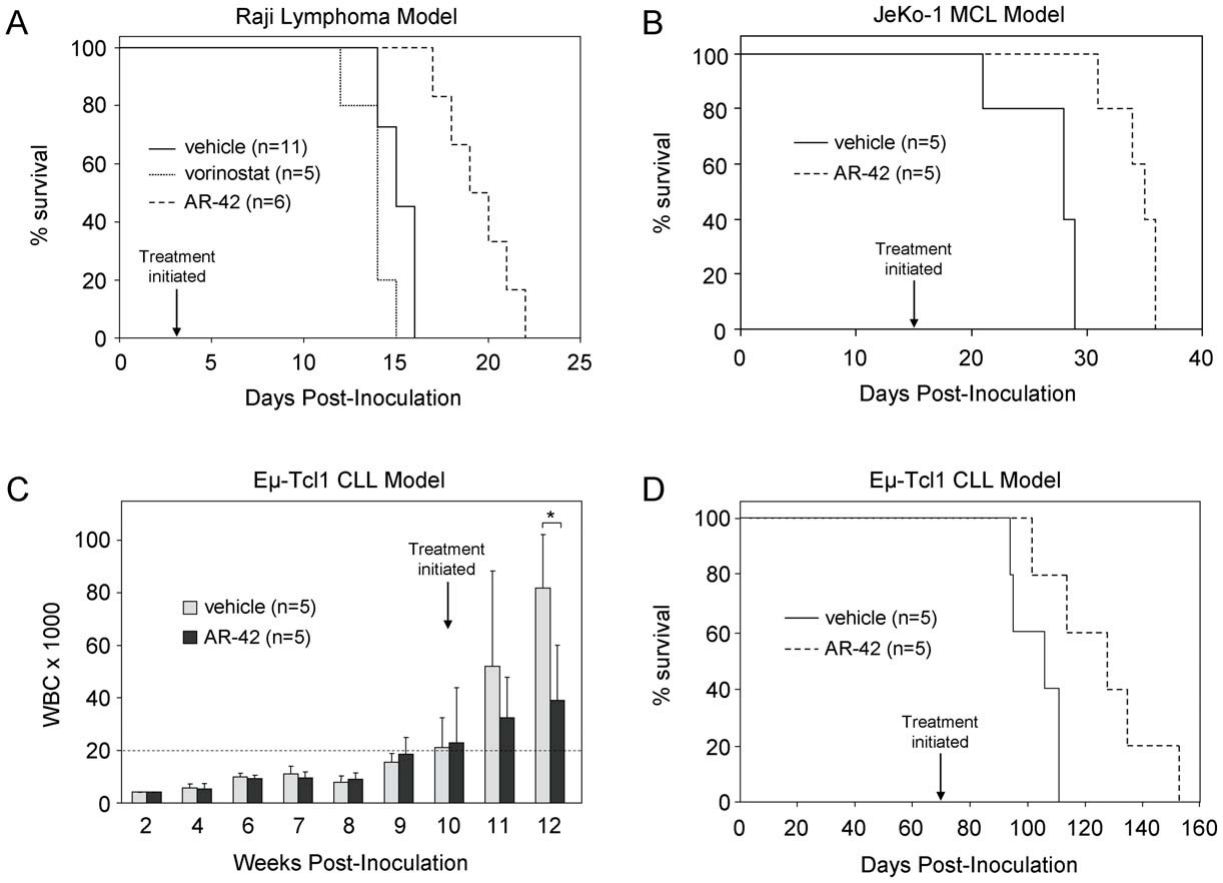
AR-42 shows *in vivo* efficacy in multiple models of B-cell malignancy. (**A**) SCID mice were engrafted with two million Raji cells via tail vein injection. Starting 3 days post-inoculation, mice were treated by oral gavage with vehicle only daily (n = 11), vorinostat at the maximum tolerated dose of 50 mg/kg daily (n = 5), and AR-42 at 75 mg/kg (n = 6) Monday, Wednesday and Friday. The median survival advantage in the AR-42 group relative to the vehicle control group was significant (p = 0.001). (**B**) SCID mice were engrafted with forty million JeKo-1 cells via tail vein injection. Starting on day 15 post-inoculation, mice were treated intraperitoneally with vehicle alone or 20 mg/kg AR-42 every three days (n = 5 per group). The improvement in median survival relative to control was significant (p = 0.003). (**C**) SCID mice were engrafted with one million Eμ-Tcl1 leukemia cells via tail vein injection. When the circulating leukocyte count averaged 20,000 cells/µL across the group (dashed horizontal line; 69 days post-inoculation), mice were randomized to two cohorts and treated Monday, Wednesday and Friday for two weeks with vehicle only (light bars) or with AR-42 at 75 mg/kg via oral gavage (dark bars) (n = 5, both groups). *The decrease in circulating leukocytes in AR-42 treated mice vs. controls at week 12 relative to week 10 was significant (p = 0.0007). (**D**) Animals in (**C**) were followed for survival (n = 5 per group). The increase in median survival in the AR-42 group relative to control was significant (p = 0.025).

Given the promising pre-clinical data with AR-42 in CLL and transformed B-leukemia cells, we sought to determine its *in vivo* activity in this class of malignancies. Engraftment of the Raji lymphoblastic lymphoma cell line into C.B-17 SCID mice produces an aggressive disseminated B-cell lymphoma that results in hind-limb paralysis requiring euthanasia approximately 15 days post-inoculation [38]. SCID animals received two million Raji cells by tail vein injection, then were followed for three days prior to initiating treatment with AR-42, vorinostat, or vehicle control by oral gavage (**figure 5A**; n = 11 for vehicle, n = 5 for vorinostat, and n = 6 for AR-42 treatment). The median survival after the initiation of therapy was 16 days (95% CI 15–18; CV = 11%) for mice treated with AR-42 (75 mg/kg Mon-Wed-Fri), vs. 12 days (95% CI 11–14; CV = 10%) for the control group, resulting in a 33% increase in median survival (p = 0.001). In contrast, treatment with the maximum tolerated dose of vorinostat in this model (50 mg/kg every day) produced no survival benefit relative to vehicle control animals.

Following this result, we evaluated the *in vivo* activity of AR-42 in an additional lymphoma model. The JeKo-1 MCL cell line [39], [40] and its *in vivo* tumorigenicity has been previously described [40], [41]. Here, SCID mice engrafted with 40 million JeKo-1 cells by tail vein injection were treated starting day 15 post-inoculation either with AR-42 (20 mg/kg) or vehicle every third day by intraperitoneal injection (n = 5 per group). Mice receiving AR-42 showed a median survival of 20 days after the initiation of treatment (95% CI 16–21; CV = 11%), compared to 13 days (95% CI 6–14; CV = 13%) for the control group (p = 0.003; **figure 5B**).

These studies in two aggressive models of human B-cell lymphoma demonstrate the *in vivo* activity of AR-42 in B-cell lymphoproliferative disorders. To explore the effects of AR-42 in a more indolent leukemia, we utilized the Eμ-Tcl1 transgenic leukemia mouse model previously described [42]. These mice develop disease very similar to that of CLL patients, including chronic B-leukemic disease progression, elevated Igκ^+^ B cells, splenomegaly, and infiltration of B-lymphocytes to the liver, lungs, and kidney [42], [43]. We employed a transplant model, in which one million leukocytes from the enlarged spleen of a leukemic Eμ-Tcl1 mouse were injected into a cohort of C.B-17 SCID mice via tail vein, essentially as described by Wu *et al*. [44]. Treatment was initiated when leukemia was evident by a peripheral leukocyte count of 20,000/µL averaged across the group (range 10,000–60,000) and palpable spleens, which occurred at week 10 following inoculation. At this point, mice were treated with vehicle or 75 mg/kg AR-42 (n = 5 per group) Monday, Wednesday and Friday for two weeks by oral gavage. AR-42 treatment resulted in a significant reduction in peripheral blood lymphocytes, examined two weeks after therapy initiation, relative to control mice (p = 0.0007; **figure 5C**). Leukemic mice treated with AR-42 also had a significant survival advantage over vehicle-treated controls (p = 0.0251) with a median survival of 58 days (95% CI: 32–83; CV = 34%) after the initiation of therapy, compared to 37 days (95% CI: 25–42; CV = 25%) in the control group (**figure 5D**). These studies utilizing three murine models of different types of B-cell lymphoma collectively demonstrate *in vivo* activity of AR-42.

## Discussion

AR-42 is a novel class I and II DAC inhibitor that has shown pre-clinical activity in a variety of solid tumor *in vitro* and *in vivo* models [19]. Here, we demonstrate that AR-42 has potent *in vitro* and *in vivo* activity in multiple models of human B-cell malignancy and provide data supporting its clinical development in this group of diseases. Unlike other compounds whose efficacy is influenced by human serum protein binding (*e.g*. flavopiridol), we found that AR-42 has similar cytotoxic effect regardless of whether human or bovine serum matrices are used. Importantly, we demonstrate that AR-42 efficacy in CLL cells is not compromised by co-culture with stromal cells, which have been widely shown to prevent spontaneous apoptosis and mediate drug resistance in CLL tumor cells [45]. We validate the class I and class II DAC specificity of AR-42 by demonstrating it promotes acetylation of histones and of tubulin at concentrations that promote cytotoxicity in B-leukemia cells, indicating its ability to inhibit both classes of DACs at biologically relevant concentrations. AR-42 induces caspase-dependent cell death, as cytotoxicity can be blocked by caspase inhibition, although details of this mechanism remain to be investigated. As shown with other DAC inhibitors, AR-42 augments the cytotoxic activity of TRAIL in CLL cells. This is potentially due to reduction of c-FLIP protein, an effect we previously reported in CLL cells using romidepsin [10]. A study in colon cancer cell lines [46] showed that the DAC inhibitor sodium butyrate also caused substantial decrease in c-FLIP protein concurrent with TRAIL sensitization, although similar studies in several hematological cell lines using sodium butyrate and vorinostat demonstrated TRAIL sensitization without reduction of c-FLIP [34]. The reason for differences in c-FLIP expression in various cell types following DAC inhibitor treatment and the importance of this in TRAIL sensitization remains unclear, although antibody reagent differences must be considered as reported here and also by Inoue *et al*. [36]. Identifying biological reasons for c-FLIP changes may shed light on the qualitative as well as quantitative differences of the various DAC inhibitors, and may guide future combination strategies. Regardless, these results suggest the involvement of both the intrinsic and extrinsic pathways of apoptosis in AR-42-mediated cytotoxicity in B-cells.

Importantly, AR-42 demonstrates *in vivo* activity in murine models of Burkitt's lymphoma, MCL, and CLL. With all three models, increased survival with AR-42 is observed compared to the vehicle control. Interestingly, in the Raji Burkitt's lymphoma model, the class I/II DAC inhibitor vorinostat administered at its maximum tolerated dose (MTD) lacked activity, whereas AR-42 showed statistically significant activity without discernable toxicity. It should be noted that our selection of doses of each agent were based on MTD in SCID mice as determined by weight loss greater than 20%. We acknowledge that direct comparison of AR-42 and vorinostat *in vivo*, even within the same model, is complicated by potentially differing pharmacologic properties such as oral absorption and half-life, as well as toxicities unrelated to weight loss. Thus it remains to be determined whether this difference in efficacy will be observed in leukemia patients. However, these data collectively support future clinical development of AR-42 in the treatment of lymphoid malignancies.

An important consideration with DAC inhibitors in the treatment of hematologic malignancies is the development of combination strategies with other targeted therapies. As has been reported with other DAC inhibitors, AR-42 significantly sensitizes CLL patient cells to TRAIL. This finding is important, as TRAIL alone has little activity in CLL but also shows little or no toxicity toward non-tumor cells. Thus, the combination of AR-42 and TRAIL receptor agonists may provide improved clinical benefit without substantial side effects. In particular, antibodies targeting DR5 are quite attractive, as they have shown extended half-life.

The importance of dual inhibition of DAC classes I and II is unclear. Most investigations of DAC inhibitors in B-cell malignancies have used class I-specific inhibitors (*e.g*. romidepsin [11], entinostat [47], and MGCD-0103 [48]). Clinical results in B-cell diseases using each of these agents have been disappointing to date, although vorinostat and romidepsin show significant activity in cutaneous T-cell lymphoma and are FDA-approved for this purpose. Using microarray analysis of CEM T-cell lymphoma cells treated with vorinostat versus romidepsin [49], Peart *et al*. determined that the pattern of gene expression is largely similar between these two groups. In addition we observed no cytotoxic effect of DAC6 inhibition in CLL patient cells, suggesting that acetylation of tubulin and/or HSP90 is not required for DAC inhibitor-mediated cytotoxicity in these cells. However AR-42 may influence other pathways controlled by class II DACs, although these are not well defined. For example, class II DACs can function as transcriptional co-repressors, and it is possible that inhibition of these enzymes allows expression of genes with pro-apoptotic effects [50]. Based on the results presented here and our previous experience with class I-specific DAC inhibitors in B-cell malignancies, we hypothesize that the more potent dual inhibition of class I and II DACs allowed by AR-42 relative to other available agents will produce clinical efficacy in B-cell leukemias including CLL.

A major question arising from work with DAC inhibitors in CLL and related B-cell lymphoid malignancies is whether there is sufficient justification to pursue this class of drugs clinically. As noted above, clinical investigations of DAC inhibitors in B-cell malignancies have shown only modest activity. Romidepsin produced a reduction in leukemic cell count in patients with advanced CLL, but without partial or complete responses by NCI criteria [11]. Similarly, MGCD0103 was also studied in a phase II trial including patients with relapsed CLL in which no clinical responses were observed in 21 patients [15]. In both studies, significant fatigue and constitutional symptoms limited patient willingness to continue therapy beyond 1–2 monthly treatments. MGCD0103 has evidence of activity in other types of lymphoma, as demonstrated by a preliminary phase II study of 38 patients in which four responses were reported among follicular lymphoma and large cell lymphoma subtypes [51]. Also, in Hodgkin's disease a 40% response rate was observed among relapsed and refractory patients [52]. After a temporary hold to investigate pericarditis in a subset of patients, clinical development of MGCD0103 continues.

In contrast to these class I DAC-specific agents, clinical investigation of class I/II DAC inhibitors in B-cell malignancies has been extremely limited. Vorinostat showed moderate activity (four complete or partial responses out of 30 B-cell lymphomas) in a study population that included multiple types of leukemia/lymphoma [53]. A phase II study of vorinostat in different types of low-grade NHL demonstrated a 37% response rate in follicular lymphoma and marginal zone lymphoma [54]. In a preliminary report, the class I/II DAC inhibitor panobinostat (LBH589) produced a 38% objective response rate in Hodgkin's disease [55]. To date, efficacy data of class I/II DAC inhibitors in CLL is nearly non-existent, with just four CLL patients treated with vorinostat as part of dose-escalation study in multiple types of leukemia [56]. Nonetheless, the encouraging *in vitro* and *in vivo* results reported here and elsewhere with class I/II DAC inhibitors in B-cell malignancies indicate that broader clinical exploration of these agents is warranted.

Previous work by members of our group [19] and studies described herein suggest that AR-42 has greater efficacy *in vitro* as well as *in vivo* compared to vorinostat. These observations suggest an improved potency and therapeutic index of AR-42 that will be of key importance in the development of this agent, given the widely observed constitutional symptoms observed with this class of drugs. Also, AR-42 shares with vorinostat and panobinostat the favorable property of oral availability, allowing far greater feasibility and flexibility of administration. Pre-clinical pharmacology and toxicology also support clinical development (data not shown), and an investigational new drug application has been approved for a first-in-man study of AR-42. Based on these collective findings, a phase I clinical trial of AR-42 in patients with B-cell lymphoid malignancies including CLL is now underway.

## Materials and Methods

### Ethics statement

Blood was obtained from CLL patients after obtaining written, informed consent according to an Ohio State University Institutional Review Board-approved protocol, in agreement with the principles of the Declaration of Helsinki. All animal research was reviewed and approved by The Ohio State University Institutional Animal Care and Use Committee.

### Patients, cell separation, and culture conditions

All patients previously received a diagnosis of CLL as defined by National Cancer Institute (NCI) criteria, had elevated leukocyte counts (>30,000 cells/µL), and were without treatment for at least four weeks prior to blood collection. CD19-positive cells were isolated from peripheral blood using Rosette-Sep reagents (StemCell Technologies, Vancouver BC) and isolated by density gradient centrifugation (Ficoll-Paque Plus; Pharmacia, Piscataway, NJ). 697 cells were obtained from DSMZ (Braunschwieg, Germany). Raji and HS-5 [27] cell lines were obtained from ATCC (Manassas, VA). The JeKo-1 MCL line [39], [40] was the gift of Dr. Raymond Lai (University of Alberta, Alberta, Canada). All cells were cultured in RPMI 1640 supplemented with 10% heat-inactivated fetal bovine serum (FBS), 100 U/ml penicillin and 100 µg/ml streptomycin (Sigma, St. Louis, MO), and 2 mM L-glutamine (Life Technologies, Grand Island, NY), at 37°C and 5% CO_2_.

### DAC inhibitors and other reagents

Romidepsin (depsipeptide) was obtained from the NCI. Tubacin was the kind gift of Drs. Ralph Mazitschek and Stuart Schreiber, The Broad Institute and Harvard University, Cambridge MA. Vorinostat and AR-42 were synthesized in the laboratory of Dr. Ching-Shih Chen, OSU College of Pharmacy. Recombinant human TRAIL/Apo2L, used at 100 ng/mL, was obtained from Cell Sciences, Inc. (Canton, MA).

### Viability assays

MTT (3-[4,5-Dimethylthiazol-2-yl]-2,5-diphenyl-tetrazolium bromide; Sigma) assays were performed as described [10]. Cells were incubated with or without drug for various times, and MTT was added. Plates were incubated for an additional 24 hr before processing and measuring by spectrophotometry. LC_50_ and IC_50_ values were calculated using Prism software (GraphPad, San Diego, CA).

### Apoptosis and flow cytometric studies

After exposure to AR-42, cells were resuspended in buffer containing annexin V-FITC and propidium iodide (PI) according to the supplier's instructions (BD Biosciences, San Diego, CA). Annexin binding and PI positivity were assessed by flow cytometry on a Coulter EPICS-XL. For caspase inhibition, 100 µM Z-VAD-fmk (benzyloxycarbonyl valine-alanine-asparagine-fluoromethyl ketone; MP Biomedicals, Aurora, OH) was added to cultures 15 minutes prior to drug addition.

### Protein and mRNA quantification

Cell extracts were prepared as previously described [10]. Total protein in each sample was quantified using the BCA protein assay (Pierce, Rockford, IL). Protein samples were separated along with molecular weight markers (BioRad, Hercules, CA) by SDS-PAGE and transferred onto nitrocellulose. Gel loading equivalence was confirmed by Ponceau S staining (Sigma) of membranes and by probing membranes with a monoclonal specific antibody for glyceraldehyde 3-phosphate dehydrogenase (GAPDH; #MAB374, Millipore, Temecula CA). Blots were incubated with chemiluminescent substrate (Pierce Super-Signal, Pierce) and exposed to x-ray film or a ChemiDoc digital imaging system (BioRad). Antibodies used were: acetylated histone H3 (#06-599, Millipore), acetylated tubulin (#T7451, Sigma), Bcl-2 (#MO887, Dako, Carpinteria, CA), polyADP-ribose polymerase (PARP) (#AM30, EMD Biosciences, La Jolla, CA), and c-FLIP (#ALX-804-428; Enzo Life Sciences, Plymouth Meeting PA). Real-time RT-PCR was performed and analyzed as described [57] using reagents, instruments and software from Applied Biosystems (Foster City, CA).

### *In vivo* studies

The use of C.B-17 SCID mice (Taconic Farm, Germantown, NY) as a lymphoma model has been described [38]. For cell line engraftments, aliquots from the same culture of cells were cryopreserved to ensure consistency of engraftments. Before inoculation, cells were thawed and cultured for 10 days. Viability was checked before engraftment to ensure greater than 90% viability.

#### Raji Engraftment Model

Cells were resuspended at 10^7^ cells/ml in PBS at room temperature, and 2×10^6^ cells were inoculated via tail vein. Treatment began 3 days after engraftment. AR-42 and vorinostat were dissolved in vehicle (0.5% methylcellulose w/v, 0.1% Tween 80 v/v, in sterile water). In pilot studies, the maximum tolerated dose (MTD) of AR-42 and vorinostat in these mice was determined to be 75 mg/kg and 50 mg/kg, respectively, when administered daily by oral gavage. MTD was defined as the maximum dose resulting in weight loss of less than 20% over the course of treatment. After engraftment, mice were randomly placed into three groups that received the following treatments: (a) vehicle alone, (b) AR-42 at 75 mg/kg every other day, (c) vorinostat at 50 mg/kg daily. Mice received treatment by oral gavage (10 µl/g body weight) for the duration of the study. The mice were monitored daily and were sacrificed if hind-limb paralysis, respiratory distress, or weight loss greater than 20% was observed. Survival (absence of the above criteria) was used as an endpoint for this study.

#### Mantle cell lymphoma model

Similar to the previous models, 6–8 week old female C.B-17 SCID mice were used. Mice were depleted of murine NK cells with intraperitoneal injections of 0.2 mg rat anti-mouse interleukin 2 (IL-2) receptor β monoclonal antibodies (TMβ1), one day before engraftment and then every week, as described [58]. Intravenous injection (in 200 µl of sterile PBS) of 4.0×10^7^ JeKo-1 cells results in a disseminated tumor after 3–4 weeks post injection and, without intervention, mice have a mean survival of 28 days [40], [41]. Starting 15 days post-injection with JeKo-1 cells, a time when established tumor burden can be documented in sentinel animals, mice (5 per group) received vehicle alone (64% saline, 12% ethanol, 24% PEG-400) or AR-42 at 20 mg/kg every three days via intraperitoneal injection. The end point of the study was survival as defined for the Raji SCID model.

#### Eμ-Tcl1 engraftment model

Development and validation of the Eμ-Tcl1 transgenic mouse as a CLL model has been described [42], [43]. An animal with a leukocyte count greater than 100,000/µl and with palpable splenomegaly was selected as a donor for engraftment. Leukocytes were recovered from the spleen of the donor, and one million cells were engrafted into C.B-17 SCID mice (female, age 6–8 weeks) via tail vein injection. Mice were randomly placed into (a) vehicle alone, or (b) 75 mg/kg AR-42 groups. Disease progression was monitored by peripheral leukocyte count using blood smears in duplicate, read by workers blinded to treatment group. Treatment began when both groups reached an average of 20,000 cells/ml. AR-42 was administered orally Monday, Wednesday, Friday for 2 weeks. Survival as noted above was used as the endpoint for evaluation.

### Statistics

To test for differences between AR-42-treated cells in the presence or absence of Z-VAD-fmk, a linear mixed effects model was used to account for dependencies among samples from the same patient. Main effects and differences were estimated from this model. Linear mixed effect models were also used to test for significant interactions between AR-42 and TRAIL. For assessments of the effect of AR-42 pretreatment in CLL cells alone or co-cultured with HS5 cells and differences in tumor load in Eμ-TCL1 mice, outcomes were natural log-transformed to stabilize variabilities among conditions and mixed effects models were then applied to the data. From these models, relevant estimates with 95% confidence intervals were obtained. For survival assessments, Kaplan-Meier estimates of the survival function for control and AR-42-treated mice were generated. Median survival times with 95% confidence intervals were calculated, and the log-rank test was used to compare the overall survival between the two groups. *P* values of less than 0.05 were considered significant. All analyses were performed using SAS/STAT software, Version 9.2 (SAS Institute Inc., Cary, NC).

## References

[1] Haggarty SJ, Koeller KM, Wong JC, Grozinger CM, Schreiber SL (2003). Domain-selective small-molecule inhibitor of histone deacetylase 6 (HDAC6)-mediated tubulin deacetylation.. Proc Natl Acad Sci U S A.

[2] Bali P, Pranpat M, Bradner J, Balasis M, Fiskus W (2005). Inhibition of histone deacetylase 6 acetylates and disrupts the chaperone function of heat shock protein 90: a novel basis for antileukemia activity of histone deacetylase inhibitors.. J Biol Chem.

[3] Kovacs JJ, Murphy PJ, Gaillard S, Zhao X, Wu JT (2005). HDAC6 regulates Hsp90 acetylation and chaperone-dependent activation of glucocorticoid receptor.. Mol Cell.

[4] Bolden JE, Peart MJ, Johnstone RW (2006). Anticancer activities of histone deacetylase inhibitors.. Nat Rev Drug Discov.

[5] Lee MJ, Kim YS, Kummar S, Giaccone G, Trepel JB (2008). Histone deacetylase inhibitors in cancer therapy.. Curr Opin Oncol.

[6] Stimson L, Wood V, Khan O, Fotheringham S, La Thangue NB (2009). HDAC inhibitor-based therapies and haematological malignancy.. Ann Oncol.

[7] Piekarz RL, Frye R, Turner M, Wright JJ, Allen SL (2009). Phase II multi-institutional trial of the histone deacetylase inhibitor romidepsin as monotherapy for patients with cutaneous T-cell lymphoma.. J Clin Oncol.

[8] Olsen EA, Kim YH, Kuzel TM, Pacheco TR, Foss FM (2007). Phase IIb multicenter trial of vorinostat in patients with persistent, progressive, or treatment refractory cutaneous T-cell lymphoma.. J Clin Oncol.

[9] Zelenetz AD (2006). Mantle cell lymphoma: an update on management.. Ann Oncol.

[10] Aron JL, Parthun MR, Marcucci G, Kitada S, Mone AP (2003). Depsipeptide (FR901228) induces histone acetylation and inhibition of histone deacetylase in chronic lymphocytic leukemia cells concurrent with activation of caspase 8-mediated apoptosis and down-regulation of c-FLIP protein.. Blood.

[11] Byrd JC, Marcucci G, Parthun MR, Xiao JJ, Klisovic RB (2005). A phase 1 and pharmacodynamic study of depsipeptide (FK228) in chronic lymphocytic leukemia and acute myeloid leukemia.. Blood.

[12] Byrd JC, Shinn C, Ravi R, Willis CR, Waselenko JK (1999). Depsipeptide (FR901228): a novel therapeutic agent with selective, in vitro activity against human B-cell chronic lymphocytic leukemia cells.. Blood.

[13] Lagneaux L, Gillet N, Stamatopoulos B, Delforge A, Dejeneffe M (2007). Valproic acid induces apoptosis in chronic lymphocytic leukemia cells through activation of the death receptor pathway and potentiates TRAIL response.. Exp Hematol.

[14] Lucas DM, Davis ME, Parthun MR, Mone AP, Kitada S (2004). The histone deacetylase inhibitor MS-275 induces caspase-dependent apoptosis in B-cell chronic lymphocytic leukemia cells.. Leukemia.

[15] Blum KA, Advani A, Fernandez L, Van Der Jagt R, Brandwein J (2009). Phase II study of the histone deacetylase inhibitor MGCD0103 in patients with previously treated chronic lymphocytic leukaemia.. Br J Haematol.

[16] Lu Q, Wang DS, Chen CS, Hu YD (2005). Structure-based optimization of phenylbutyrate-derived histone deacetylase inhibitors.. J Med Chem.

[17] Lu Q, Yang YT, Chen CS, Davis M, Byrd JC (2004). Zn2+-chelating motif-tethered short-chain fatty acids as a novel class of histone deacetylase inhibitors.. J Med Chem.

[18] Kisseberth WC, Murahari S, London CA, Kulp SK, Chen CS (2008). Evaluation of the effects of histone deacetylase inhibitors on cells from canine cancer cell lines.. Am J Vet Res.

[19] Kulp SK, Chen CS, Wang DS, Chen CY (2006). Antitumor effects of a novel phenylbutyrate-based histone deacetylase inhibitor, (S)-HDAC-42, in prostate cancer.. Clin Cancer Res.

[20] Lu YS, Kashida Y, Kulp SK, Wang YC, Wang D (2007). Efficacy of a novel histone deacetylase inhibitor in murine models of hepatocellular carcinoma.. Hepatology.

[21] Sargeant AM, Rengel RC, Kulp SK, Klein RD, Clinton SK (2008). OSU-HDAC42, a histone deacetylase inhibitor, blocks prostate tumor progression in the transgenic adenocarcinoma of the mouse prostate model.. Cancer Res.

[22] Lin TY, Fenger J, Murahari S, Bear MD, Kulp SK

[23] Byrd JC, Lin TS, Dalton JT, Wu D, Phelps MA (2007). Flavopiridol administered using a pharmacologically derived schedule is associated with marked clinical efficacy in refractory, genetically high-risk chronic lymphocytic leukemia.. Blood.

[24] Mudry RE, Fortney JE, York T, Hall BM, Gibson LF (2000). Stromal cells regulate survival of B-lineage leukemic cells during chemotherapy.. Blood.

[25] Nishio M, Endo T, Tsukada N, Ohata J, Kitada S (2005). Nurselike cells express BAFF and APRIL, which can promote survival of chronic lymphocytic leukemia cells via a paracrine pathway distinct from that of SDF-1alpha.. Blood.

[26] Panayiotidis P, Jones D, Ganeshaguru K, Foroni L, Hoffbrand AV (1996). Human bone marrow stromal cells prevent apoptosis and support the survival of chronic lymphocytic leukaemia cells in vitro.. Br J Haematol.

[27] Roecklein BA, Torok-Storb B (1995). Functionally distinct human marrow stromal cell lines immortalized by transduction with the human papilloma virus E6/E7 genes.. Blood.

[28] Medina V, Edmonds B, Young GP, James R, Appleton S (1997). Induction of caspase-3 protease activity and apoptosis by butyrate and trichostatin A (inhibitors of histone deacetylase): dependence on protein synthesis and synergy with a mitochondrial/cytochrome c-dependent pathway.. Cancer Res.

[29] Mitsiades N, Mitsiades CS, Richardson PG, McMullan C, Poulaki V (2003). Molecular sequelae of histone deacetylase inhibition in human malignant B cells.. Blood.

[30] Hideshima T, Bradner JE, Wong J, Chauhan D, Richardson P (2005). Small-molecule inhibition of proteasome and aggresome function induces synergistic antitumor activity in multiple myeloma.. Proc Natl Acad Sci U S A.

[31] Hubbert C, Guardiola A, Shao R, Kawaguchi Y, Ito A (2002). HDAC6 is a microtubule-associated deacetylase.. Nature.

[32] Kawada J, Zou P, Mazitschek R, Bradner JE, Cohen JI (2009). Tubacin kills Epstein-Barr virus (EBV)-Burkitt lymphoma cells by inducing reactive oxygen species and EBV lymphoblastoid cells by inducing apoptosis.. J Biol Chem.

[33] Inoue H, Shiraki K, Ohmori S, Sakai T, Deguchi M (2002). Histone deacetylase inhibitors sensitize human colonic adenocarcinoma cell lines to TNF-related apoptosis inducing ligand-mediated apoptosis.. Int J Mol Med.

[34] Rosato RR, Almenara JA, Dai Y, Grant S (2003). Simultaneous activation of the intrinsic and extrinsic pathways by histone deacetylase (HDAC) inhibitors and tumor necrosis factor-related apoptosis-inducing ligand (TRAIL) synergistically induces mitochondrial damage and apoptosis in human leukemia cells.. Mol Cancer Ther.

[35] Inoue S, Mai A, Dyer MJ, Cohen GM (2006). Inhibition of histone deacetylase class I but not class II is critical for the sensitization of leukemic cells to tumor necrosis factor-related apoptosis-inducing ligand-induced apoptosis.. Cancer Res.

[36] Inoue S, MacFarlane M, Harper N, Wheat LM, Dyer MJ (2004). Histone deacetylase inhibitors potentiate TNF-related apoptosis-inducing ligand (TRAIL)-induced apoptosis in lymphoid malignancies.. Cell Death Differ.

[37] MacFarlane M, Harper N, Snowden RT, Dyer MJ, Barnett GA (2002). Mechanisms of resistance to TRAIL-induced apoptosis in primary B cell chronic lymphocytic leukaemia.. Oncogene.

[38] Cattan AR, Douglas E (1994). The C.B.17 scid mouse strain as a model for human disseminated leukaemia and myeloma in vivo.. Leuk Res.

[39] Amin HM, McDonnell TJ, Medeiros LJ, Rassidakis GZ, Leventaki V (2003). Characterization of 4 mantle cell lymphoma cell lines.. Arch Pathol Lab Med.

[40] Jeon HJ, Kim CW, Yoshino T, Akagi T (1998). Establishment and characterization of a mantle cell lymphoma cell line.. Br J Haematol.

[41] Liu Q, Alinari A, Chen C-S, Yan F, Dalton JT (2010). FTY720 demonstrates promising in-vitro and in-vivo Pre-clinical Activity by Down-modulating Cyclin D1 and phospho-Akt in Mantle Cell Lymphoma.. Clinical Cancer Research (in press).

[42] Bichi R, Shinton SA, Martin ES, Koval A, Calin GA (2002). Human chronic lymphocytic leukemia modeled in mouse by targeted TCL1 expression.. Proc Natl Acad Sci U S A.

[43] Johnson AJ, Lucas DM, Muthusamy N, Smith LL, Edwards RB (2006). Characterization of the TCL-1 transgenic mouse as a preclinical drug development tool for human chronic lymphocytic leukemia.. Blood.

[44] Wu QL, Buhtoiarov IN, Sondel PM, Rakhmilevich AL, Ranheim EA (2009). Tumoricidal effects of activated macrophages in a mouse model of chronic lymphocytic leukemia.. J Immunol.

[45] Kurtova AV, Balakrishnan K, Chen R, Ding W, Schnabl S (2009). Diverse marrow stromal cells protect CLL cells from spontaneous and drug-induced apoptosis: development of a reliable and reproducible system to assess stromal cell adhesion-mediated drug resistance.. Blood.

[46] Hernandez A, Thomas R, Smith F, Sandberg J, Kim S (2001). Butyrate sensitizes human colon cancer cells to TRAIL-mediated apoptosis.. Surgery.

[47] Kummar S, Gutierrez M, Gardner ER, Donovan E, Hwang K (2007). Phase I trial of MS-275, a histone deacetylase inhibitor, administered weekly in refractory solid tumors and lymphoid malignancies.. Clin Cancer Res.

[48] Blum KA, Advani A, Fernandez L, Van Der Jagt R, Brandwein J (2009). Phase II study of the histone deacetylase inhibitor MGCD0103 in patients with previously treated chronic lymphocytic leukaemia.. Br J Haematol.

[49] Peart MJ, Smyth GK, van Laar RK, Bowtell DD, Richon VM (2005). Identification and functional significance of genes regulated by structurally different histone deacetylase inhibitors.. Proc Natl Acad Sci U S A.

[50] Yang XJ, Seto E (2008). The Rpd3/Hda1 family of lysine deacetylases: from bacteria and yeast to mice and men.. Nat Rev Mol Cell Biol.

[51] Younes A, Wedgwood A, McLaughlin P, Andreadis C, Assouline AE (2007). Treatment of relapsed or refractory lymphoma with the oral isotype-selective histone deacetylase inhibitor MGCD0103: Interim results from a Phase II study.. Blood.

[52] Younes A, Pro B, Fanale M, McLaughlin P, Neelapu S (2007). Isotype-selective HDAC inhibitor MGCD0103 decreases serum TARC concentrations and produces clinical responses in heavily pretreated patients with relapsed classical Hodgkin Lymphoma.. Blood.

[53] O'Connor OA, Heaney ML, Schwartz L, Richardson S, Willim R (2006). Clinical experience with intravenous and oral formulations of the novel histone deacetylase inhibitor suberoylanilide hydroxamic acid in patients with advanced hematologic malignancies.. J Clin Oncol.

[54] Kirschbaum M, Popplewell L, Nademanee AP, Pullarkat V, Delioukina M (2008). A Phase 2 study of vorinostat (Suberoylanilide Hydroxamic Acid, SAHA) in relapsed or refractory indolent Non-Hodgkin's Lymphoma. A California Cancer Consortium study.. Blood.

[55] Dickinson M, Ritchie D, DeAngelo DJ, Spencer A, Ottmann OG (2009). Preliminary evidence of disease response to the pan deacetylase inhibitor panobinostat (LBH589) in refractory Hodgkin Lymphoma.. Br J Haematol.

[56] Garcia-Manero G, Yang H, Bueso-Ramos C, Ferrajoli A, Cortes J (2008). Phase 1 study of the histone deacetylase inhibitor vorinostat (suberoylanilide hydroxamic acid [SAHA]) in patients with advanced leukemias and myelodysplastic syndromes.. Blood.

[57] Lucas DM, Edwards RB, Lozanski G, West DA, Shin JD (2009). The novel plant-derived agent silvestrol has B-cell selective activity in chronic lymphocytic leukemia and acute lymphoblastic leukemia in vitro and in vivo.. Blood.

[58] Tanaka T, Kitamura F, Nagasaka Y, Kuida K, Suwa H (1993). Selective long-term elimination of natural killer cells in vivo by an anti-interleukin 2 receptor beta chain monoclonal antibody in mice.. J Exp Med.

